# Isolation and Functional Characterization of a Phenylalanine Ammonia-Lyase Gene (*SsPAL1*) from *Coleus* (*Solenostemon scutellarioides* (L.) Codd)

**DOI:** 10.3390/molecules200916833

**Published:** 2015-09-16

**Authors:** Qinlong Zhu, Xianrong Xie, Haoxiang Lin, Shunzhao Sui, Rongxin Shen, Zhongfang Yang, Kun Lu, Mingyang Li, Yao-Guang Liu

**Affiliations:** 1 State Key Laboratory for Conservation and Utilization of Subtropical Agro-Bioresources, College of Life Sciences, South China Agricultural University, Guangzhou 510642, China; E-Mails: xie079799@163.com (X.X.); lhaoxiang@163.com (H.L.); smallking@163.com (R.S.); yzfmora@163.com (Z.Y.); 2 Key Laboratory of Plant Functional Genomics and Biotechnology of Guangdong Provincial Higher Education Institutions, Guangzhou 510642, China; 3 Chongqing Engineering Research Center for Floriculture, College of Horticulture and Landscape, Southwest University, Chongqing 400716, China; E-Mails: sszcq@126.com (S.S.); limy@swu.edu.cn (M.L.); 4 Chongqing Engineering Research Center for Rapeseed, College of Agronomy and Biotechnology, Southwest University, Chongqing 400716, China; E-Mail: drlukun@swu.edu.cn

**Keywords:** phenylalanine ammonia-lyase, anthocyanin, *Coleus* (*Solenostemon scutellarioides* (L.) Codd)

## Abstract

Phenylalanine ammonia-lyase (PAL) is the first enzyme involved in the phenylpropanoid pathway and plays important roles in the secondary metabolisms, development and defense of plants. To study the molecular function of PAL in anthocyanin synthesis of *Coleus* (*Solenostemon scutellarioides* (L.) Codd), a *Coleus* PAL gene designated as *SsPAL1* was cloned and characterized using a degenerate oligonucleotide primer PCR and RACE method. The full-length *SsPAL1* was 2450 bp in size and consisted of one intron and two exons encoding a polypeptide of 711 amino acids. The deduced *SsPAL1* protein showed high identities and structural similarities with other functional plant PAL proteins. A series of putative *cis*-acting elements involved in transcriptional regulation, light and stress responsiveness were found in the upstream regulatory sequence of *SsPAL1*. Transcription pattern analysis indicated that *SsPAL1* was constitutively expressed in all tissues examined and was enhanced by light and different abiotic factors. The recombinant *SsPAL1* protein exhibited high PAL activity, at optimal conditions of 60 °C and pH 8.2. Although the levels of total PAL activity and total anthocyanin concentration have a similar variation trend in different *Coleus* cultivars, there was no significant correlation between them (*r* = 0.7529, *p* > 0.1), suggesting that PAL was not the rate-limiting enzyme for the downstream anthocyanin biosynthetic branch in *Coleus*. This study enables us to further understand the role of *SsPAL1* in the phenylpropanoid (flavonoids, anthocyanins) biosynthesis in *Coleus* at the molecular level.

## 1. Introduction

*Solenostemon scutellarioides* (L.) Codd (Lamiaceae, synonym *Coleus blumei* Benth; *Coleus scutellarioides* (L.) Benth), also known as *Coleus*, is a well-known ornamental plant that has very colorful foliage in different cultivars. It is used as a garden plant worldwide and as a medicinal plant in some countries, including India, Indonesia, Mexico, *etc.* [1]. The most prominent secondary metabolites of *Coleus* are rosmarinic acid (RA) and anthocyanins [2,3]. RA, an ester of caffeic acid with 3,4-dihydroxyphenyl lactic acid, is widespread in medicinal plants of the family Lamiaceae and synthesized by starting with the precursor molecules l-phenylalanine and l-tyrosine. RA might be involved in the plant’s defense system against fungal and bacterial infections and predators [4]. Anthocyanins, one type of flavonoids derived from a branch of the phenylpropanoid biosynthetic pathway, are the major pigments imparting a wide range of different colors from red to purple in leaves of *Coleus* [3]. Recently, genes involved in RA biosynthesis have been cloned and characterized from *Coleus* [4,5,6,7]. However, there is limited information about the genes related to the phenylpropanoid and anthocyanins pathway in this ornamental and medicinal plant.

The phenylpropanoid pathway, one of the most important secondary metabolic pathways, produces a variety of biologically-important metabolites in plants, such as flavonoids, anthocyanins and lignins [8,9]. Phenylalanine ammonia-lyase (PAL, EC 4.3.1.5), the first enzyme and also a rate-limiting step in the phenylpropanoid pathway, catalyzes the deamination of l-phenylalanine to *trans*-cinnamic acid, which is a regulation point linking primary and secondary metabolism. In addition, as the first enzyme involved in anthocyanin biosynthesis, the PAL activity is related to anthocyanin content in many plants [10]. Due to these important roles, PAL has been widely studied in many plants, including rice [11], *Arabidopsis* [12,13], loblolly pine [14], *Salvia miltiorrhiza* [15], *Lycoris radiata* [16], *Bambusa oldhamii* [17], *Juglans regia* [18], *Melissa officinalis* [19], *etc.* In most plants, *PAL* genes belong to a gene family, and each member of the PAL family shows a distinct expression pattern and activity difference. For example, in *Arabidopsis thaliana*, there are four PAL members (*AtPAL1*–*AtPLA4*): *AtPAL1* and *2* were co-expressed in different plant organs and have functional specialization in abiotic environmental-triggered flavonoid synthesis; *AtPAL3* was strongly expressed in leaves, but showed very low transcript levels in different stages of stems; *AtPAL4* was strongly expressed in inflorescent stems and was related to tissue-specific lignin synthesis. Additionally, the four isoforms have different activities: *AtPAL1*, *2* and *4* have strong catalytic activity, but *AtPAL3* almost no enzyme activity [13].

Therefore, the isolation and characterization of the PAL gene would help to explore the molecular regulatory mechanism of phenylpropanoid and anthocyanin biosynthesis in *Coleus*. In this study, we reported the cloning of a full-length PAL gene (designed as *SsPAL1*) from *Coleus* and characterized its molecular features, expression patterns and functional activities. As far as we know, this is the first PAL gene reported in *S. scutellarioides* (L.) Codd.

## 2. Results and Discussion

### 2.1. Cloning and Characterization of the Full-Length SsPAL1 cDNA Sequence

**Figure 1:**
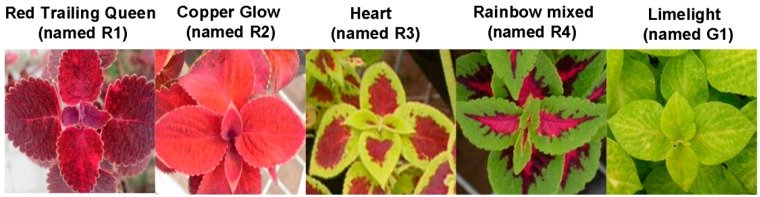
Five *Coleus* (*S. scutellarioides* (L.) Codd) lines in this study. R, red; G, green.

Using the degenerate oligonucleotide primer PCR (DOP-PCR), an 860-bp fragment was amplified from leaves of *Coleus* cultivar R1 (Red Trailing Queen; Figure 1). After cloning, sequencing and alignment, two types of different conserved fragments of PAL gene (*PAL1* and *PAL2*) were identified in 10 clones (Figure S1). The *PAL1* and *PAL2* showed 76% DNA sequence identity with each other. Between them, the fragment of *PAL1* with higher identity to plants PAL genes was used to do 5′- and 3′-RACE, and a full-length cDNA of *SsPAL1* was obtained (Figure S2).

**Figure 2:**
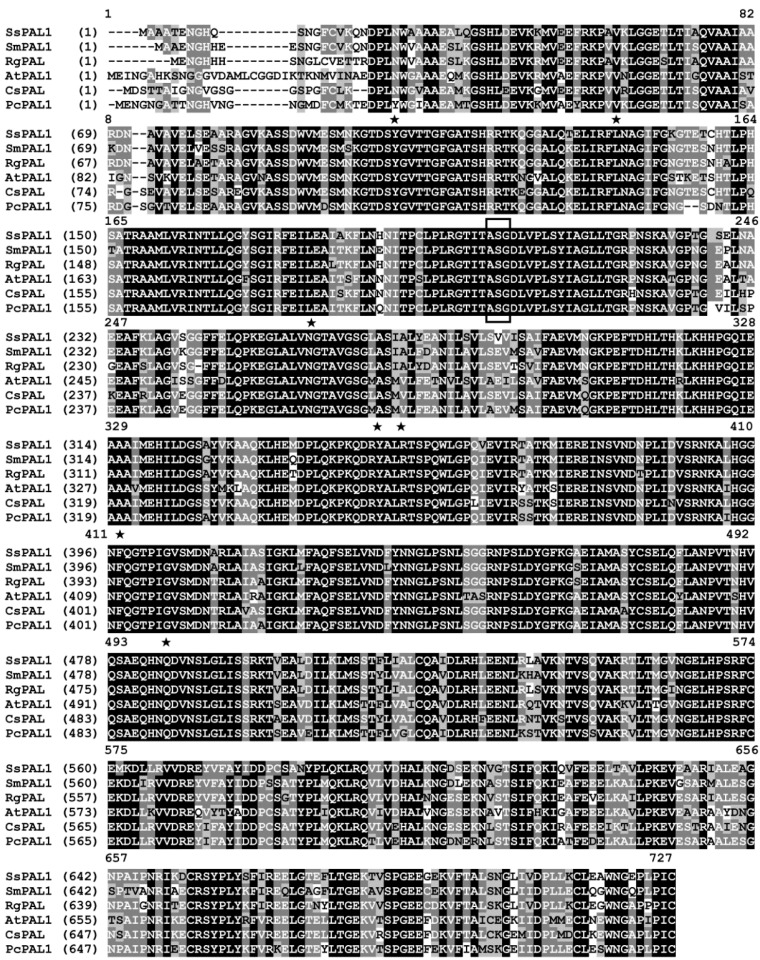
Multi-sequence alignment of *SsPAL1* with other plant PAL proteins. The sequences shown here are from *Solenostemon scutellarioides* (*SsPAL1*, JQ975419), *Salvia miltiorrhiza* (*SmPAL1*, EF462460), *Rehmannia glutinosa* (*RgPAL*, AAK84225), *Arabidopsis thaliana* (*AtPAL1*, AY303128), *Camellia sinensis* (*CsPAL*, D26596) and *Petroselinum crispum* (*PcPAL1*, CAA68938). The highly-conserved Ala-Ser-Gly triad that acts as the active site forming the MIO prosthetic group for non-oxidative deamination is in a box. The conserved active sites are shown by a five-pointed star.

The full-length cDNA is 2356 bp, containing a 95-bp 5′-UTR, a translation start site for eukaryotic genes (ATCATGG), a 2136-bp ORF encoding a 711-amino acid protein and a 125 bp 3′-UTR, including a potential polyadenylation signal AATATAA (GenBank Accession No. JQ975419) (Figure S3). A BLAST and multiple sequence alignment analysis showed that the deduced peptide sequence of *SsPAL1* has a high similarity to other known plant PALs, sharing a similarity of 90% identity to *SmPAL1* from *Salvia miltiorrhiza* (ABR14606) and *RgPAL* from *Rehmannia glutinosa* (AAK84225), 86% identity to both *ArPAL* from (AAK15640) and *PcPAL1* from *Petroselinum crispum* (CAA68938) and 80% identity to *AtPAL1* from *Arabidopsis thaliana* (AEC09341) (Figure 2).

In addition, the conserved amino acid residues in plant PALs were found in *SsPAL1*, as Y103, L131, A197, S198, G199, N255, Q342, Q342, R345, R349, F395 and Q483 (Figure S3 and Figure 2). Furthermore, a phenylalanine and histidine ammonia-lyase active site consensus sequence (G-[STG]-[LIVM]-[STG]-[AC]-S-G-[DH]-L-X-P-L-[SA]-X(2)-[SAV]) [20] was found at position 193–209, in which there was the key active site Ala-Ser-Gly (197–199) forming a 3,5-dihydro-5-methylidene-4*H*-imidazol-4-one (MIO) group [21] (Figure S3 and Figure 2).

### 2.2. Characterization of the Deduced SsPAL1 Protein

Bioinformatic analyses of deduced *SsPAL1* protein showed that *SsPAL1* has high sequence and structure conservation with known functional PAL proteins. The predicted isoelectric point (pI) and a molecular weight of *SsPAL1* were 6.52 and 76.99 kDa, respectively. Many conserved active sites and domains [22,23] were found in the deduced *SsPAL1* protein sequence (Figure 2 and Figure S3). Many possible phosphorylation sites (16 for Ser, eight for Thr and eight for Tyr) [22] were found in *SsPAL1*, suggesting that phosphorylation may play an important role for normal functioning of *SsPAL1*.

**Figure 3:**
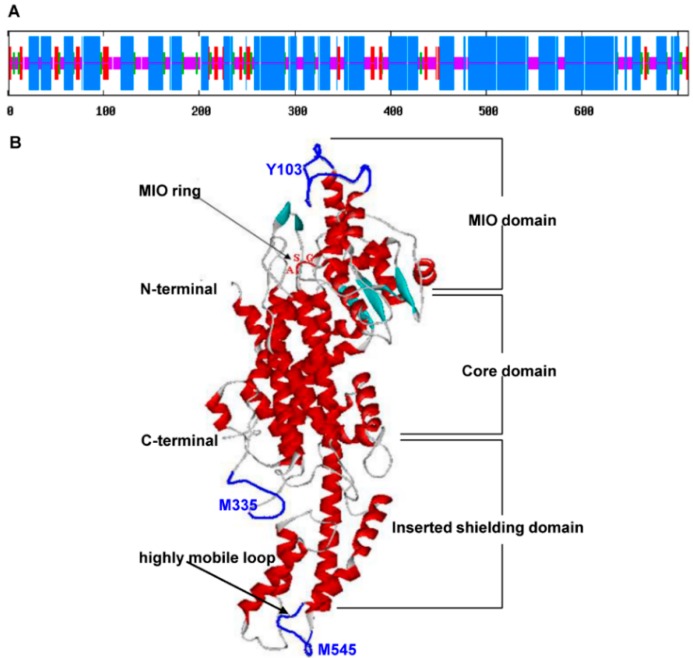
Predicted molecular model of *SsPAL1*. (**A**) Predicted secondary structure of *SsPAL1*. The red, green, blue and pink regions represent the extended strand, beta turn, alpha helix and random coil, respectively; (**B**) Predicted tertiary structure of *SsPAL1*. The α-helices, β-sheets, turns and coils are shown in red, cyan, dark and white, respectively. The MIO ring, Ala-Ser-Gly, is marked red. The highly-mobile loops at residues 103, 335 and 545 are blue.

By using the SOPMA program [24], the secondary structure prediction of *SsPAL1* revealed that α-helices (56.12%) were the main structural elements, and random coils (30.38%) were scattered in the entire protein (Figure 3A). By using the known *P. crispum* *PcPAL* crystal structure (1W27A) [23] as a template in the SWISS-MODEL program [25], the tertiary structure showed that *SsPAL1* consisted of the MIO domain, a core domain and an inserted shielding domain like a “sea-horse” shape [21,23] (Figure 3B). In the MIO domain, there was a highly-conserved Ala-Ser-Gly triad, which acts as the active site forming the MIO prosthetic group for non-oxidative deamination [26]. The highly-mobile loops at residues Y103, M335 and M545, whose mobility is required for the catalysis of PAL, were found in the tertiary structure prediction of *SsPAL1*. These data imply that *SsPAL1* might be a functional protein with catalytic activity similar to PcPAL.

### 2.3. Phylogenetic Analysis of SsPAL1

**Figure 4:**
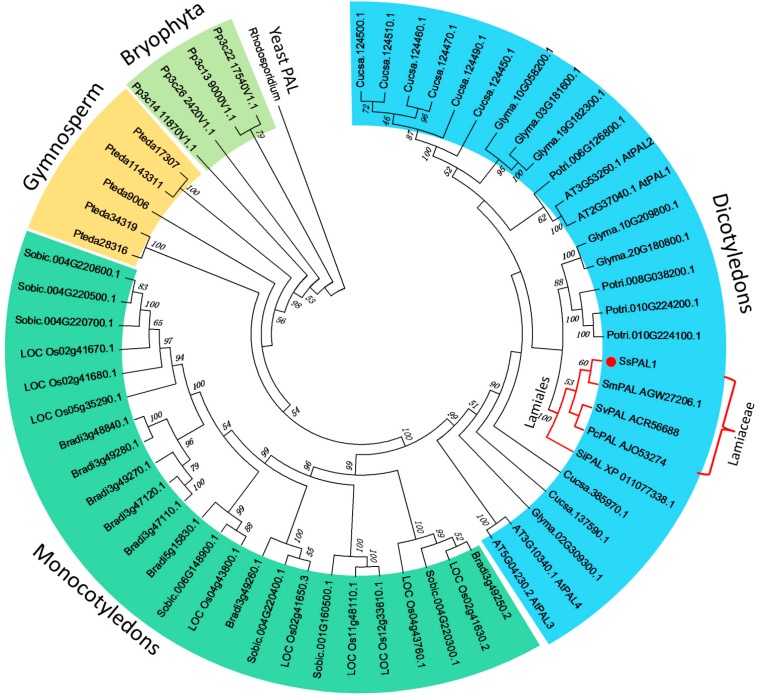
Phylogenetic tree of the plant PAL gene family. The amino acid sequences are aligned by ClustalX (1.81) and the neighbor-joining (NJ) tree as constructed using the program MEGA6.0. Numbers above branches are bootstrap values (>45%). *SsPAL1* is marked with a red dot, and the order Lamiales is marked with red lines.

To study the evolutionary relationship between *SsPAL1* and other PAL proteins, a set of PAL amino acid sequences from other plants (Table S1) were used to construct a phylogenetic tree by the neighbor-joining method using the MEGA 6.0 program (Figure 4). In general, the topology of the phylogenetic tree agrees with the traditional taxonomy classification and is similar to that obtained by Wu *et al.* (2014) [27]. On the phylogenetic tree, most of the plant PAL proteins were grouped into four branches representing dicotyledons, monocotyledons, gymnosperms and bryophytes, using a PAL (P11544) from yeast (*Rhodosporidium toruloides*) [28] as an out-group. In the angiosperm-type PAL family, dicotyledon and monocotyledon PALs independently form each of the subfamilies. *SsPAL1* is clustered together with other PAL from Lamiaceae species, including PoPAL from *Pogostemon cablin*, *SmPAL1* from *S. miltiorrhiza* [15] and *SvPAL* from *Scutellaria viscidula*. In this subgroup, the *SsPAL1* has a closer relationship to the *SmPAL1*, which is a known functional PAL gene. These results indicate that *SsPAL1* belongs to a typical PAL gene derived from Lamiaceae species and may have a similar function as *SmPAL1* in the phenylpropanoid pathway.

### 2.4. Genomic Organization of SsPAL1

**Figure 5:**
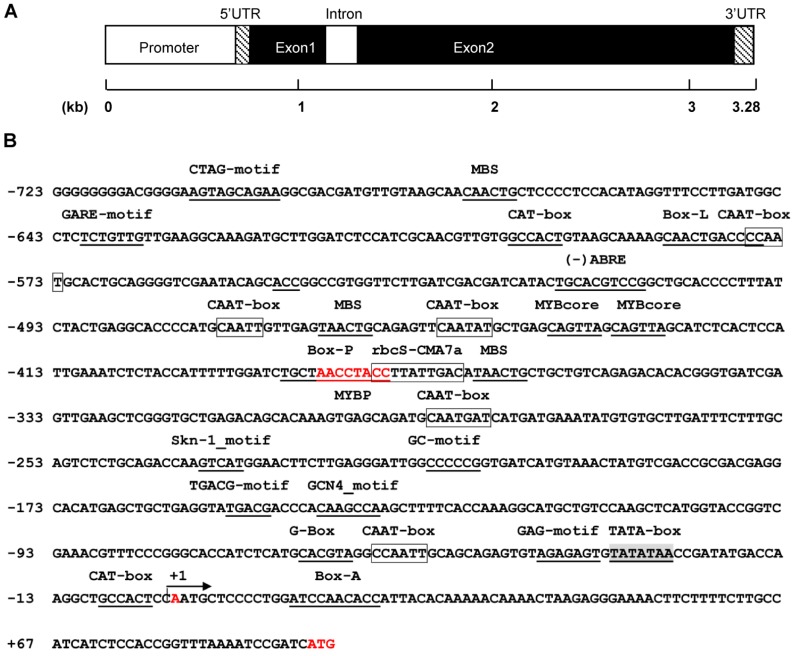
The genomic organization and the predicted *cis*-acting elements in the 5′ upstream region of *SsPAL1*. (**A**) The promoter, 5′-UTR, 3′-UTR, exons and intron are shown in the figure; (**B**) the translation start site (ATG), transcription start site (+A) and conserved MYBP motif (AACCTACC) are shown in red, respectively. The most probable TATA box is shaded and underlined. The CAAT motifs and rbcS-CMA7a are boxed and other predicted *cis*-acting elements underlined.

In order to inspect the organization of the gene, the genomic sequence of *SsPAL1* was amplified from *Coleus* genomic DNA by PCR with two specific primers Fpal1 and Rpal1 (Table S2). Sequencing analysis showed that *SsPAL1* has two exons and one intron (Figure S3 and Figure 5A). The intron of 189 bp was a typical plant intron that possesses the standard GT/AG splicing site and a higher A + T content (76.72%) than the exons (43.07%) (Figure S3). The genomic organization of *SsPAL1* gene was in accordance to the typical plant PAL genes, similar to those of *AtPAL1* and *AtPAL2* in *Arabidopsis thaliana* [12,13], *SmPAL1* in *S. miltiorrhiza* [15] and *CcPAL1* in *Coffea canephora* [20]; while it was different from *AtPAL3* and *AtPAL4*, which contained two introns and three exons, *BoPAL1* in *B. oldhamii* [17] and *GbPAL* in *Ginkgo biloba* [29], which did not have an intron. The intron of *SsPAL1* was inserted between the second and third bases of the R129 codon (Figure S3). Its location was very conservative and the same as in *AtPAL1* [12] and *SmPAL1* [15], even though the intron length varied among them.

A large number of studies have shown that *PAL* is encoded by a small gene family with 2–13 members in most plants [12,15,17,29,30,31]. In *Coleus*, two different types of *PAL* gene fragments, *PAL1* and *PAL2*, were identified by sequencing 10 clones of the *PAL* conserved fragment (Figure S1). Among them, seven clones were *PAL1* and three clones were *PAL2.* Although the *PAL2* fragment had only 76% DNA sequence identity to *PAL1*, they shared 87% sequence identity at the protein level. In addition, *Coleus* was described as a tetraploid [3]. Therefore, there were at least two PAL members in *Coleus*: one was *SsPAL1* from the conserved *PAL1* fragment; the other corresponded to the conserved *PAL2* fragment.

### 2.5. Analysis of the Upstream Region of SsPAL1

In the promoter region of eukaryotic genes, there are many important *cis*-acting elements determining gene expression pattern and level. For better understanding the biological function of *SsPAL1*, an 826-bp upstream fragment was obtained from the *Coleus* genome using the hiTAIL-PCR method. After alignment of the obtained 5′ upstream sequence and 5′-UTR region of *SsPAL1*, the overlapping sequence was found, and the transcription start site of *SsPAL1* was also identified, located 92 bp upstream from the start codon ATG (Figure 5B).

The putative *cis*-acting elements in the promoter region of *SsPAL1* were predicted using the PLACE and Plant-CARE programs. The results of the location and function of the predicted *cis*-acting elements are shown in Figure 5B and Table S3. The core promoter elements TATA-box and enhancer *cis*-acting elements CAAT-box were found in the 5′-flanking region of *SsPAL1*. The TATA box was most likely located at −32, similar to other eukaryotic genes [11]. Two typical *cis*-acting elements [15] in promoters of phenylpropanoid biosynthesis genes were also found, including a P-box at position −384 and an l-box located at −596. Multiple plant MYB binding sites were predicted in this region, especially including an MYBP motif that was a consensus sequence related to P-box in promoters of phenylpropanoid and flavonoid biosynthetic genes, such as *PAL*, *CHS*, *CHI*, *DFR*, *CL* and *Bz1* [32,33,34,35], suggesting that MYB transcription factors may be involved in the regulation of *SsPAL1* expression. In addition, many *cis*-acting elements involved in light responsiveness were found in the 5′-flanking region of *SsPAL1*, indicating that light could be an important regulator for the expression of *SsPAL1*. Besides, there were several stress and hormone-responsive elements, such as W1-box, ABRE, TGACG-motif and GARE-motif, in this region, implying that the expression of *SsPAL1* may be also regulated by wounding, pathogen infection, ABA, MeJA and GA.

### 2.6. Transcription Profiles of SsPAL1

**Figure 6:**
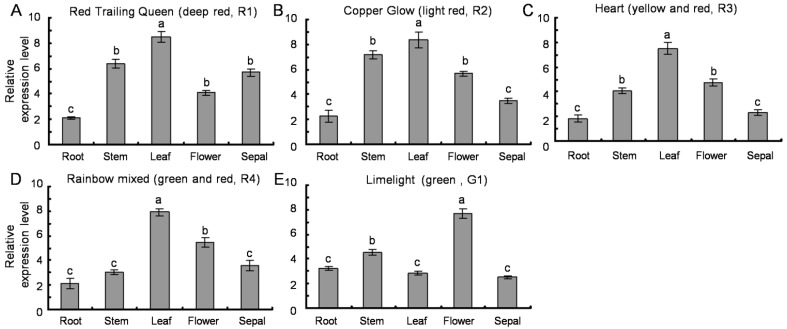
Expression pattern of *SsPAL1* in different tissues of five *Coleus* cultivars by qRT-PCR analysis (**A**–**E**). The qRT-PCR results were calculated as differences in cycle threshold (Ct) between the *SsPAL* and *SsACT* gene (2^−∆Ct^). All reactions were carried out in triplicate, and each experiment was repeated twice. The same lowercase letter is not significantly different (*p* > 0.05).

Due to the difference in anthocyanins content, the five *Coleus* cultivars (R1, R2, R3, R4 and G1) show different leaf colors. For example, the leaf of R1 is dark red with chartreuse-green edges, but G1 is only green with lemon yellow. In order to examine the expression level of *SsPAL1* in different tissues of five cultivars, the total RNAs were extracted from root, stem, leaf, flower and sepal, and the gene transcription levels were detected by qRT-PCR. Results indicated that *SsPAL1* was constitutively expressed in all examined tissues of five cultivars, and the expression level is different in examined tissues of each cultivar (Figure 6). Colored cultivars (R1–R4) have a similar expression pattern of *SsPAL1*: the highest expression level is in leaf and the lowest is in root or sepal. However, in green cultivar G1, the highest level of transcript is in flower. The different expression patterns of PAL genes also exist in others plants. In most cases, the higher levels of PAL expression are in leaves, such as in *P. crispum* [36], *S. miltiorrhiza* [15] and *G. biloba* [29]. Sometimes, PAL genes are expressed more highly in roots of tobacco [37] or in flowers of *Jatropha curcas* [32] and *Cistanche deserticola* [18].

**Figure 7:**
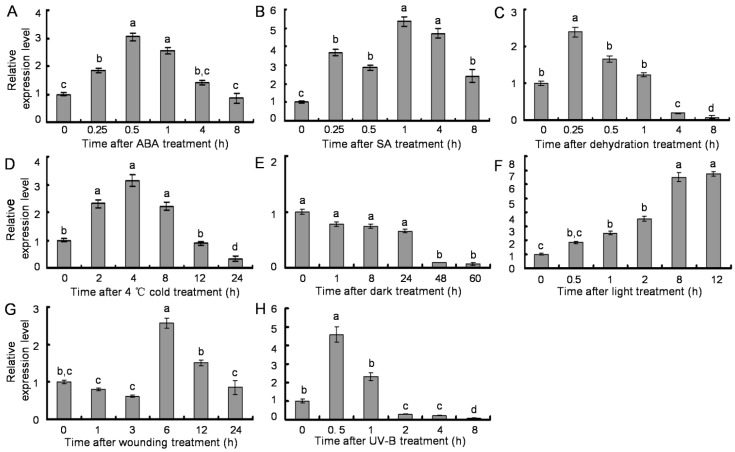
Transcription pattern of *SsPAL1* under different treatments by qRT-PCR analysis. (**A**) 100 μM ABA; (**B**) 500 μM SA; (**C**) dehydration; (**D**) 4 °C cold; (**E**) dark; (**F**) 25 °C in 24,000 Lx light; (**G**) wounding; and (**H**) 1500 μJ/m^2^ UV-B. The results were analyzed using the comparative Ct method and presented as fold changes compared to 0 h untreated leaves (2^−∆∆Ct^). All reactions were carried out in triplicate, and each experiment was repeated twice. The same lowercase letter is not significantly different (*p* > 0.05).

A number of studies have shown that PAL gene expression is induced by various environmental factors, including pathogen infection, wounding, UV irradiation and low temperatures [9]. To detect the inducible transcription pattern of *SsPAL1*, RNA samples were isolated from R1 leaves that were treated by different treatments. As shown in Figure 7, qRT-PCR results revealed that the expression profiles of *SsPAL1* were responsive to different environmental stress conditions. For ABA treatment, *SsPAL1* expression level gradually increased in the initial 0.5 h and then decreased 1.5-fold at 4 h. When the time point was up to 8 h, its expression was reduced to the initial level (Figure 7A). The transcript of *SsPAL1* was rapidly increased four-fold after SA treatment for 15 min and then declined after 0.5 h of treatment, but again increased up to the highest level at 1 h of treatment. However, with the extension of treatment time, its expression declined (Figure 7B). A high level of *SsPAL1* expression was observed within 15 min of induced treatment by dehydration and then followed by a reduction. After 8 h of treatment, the gene transcripts became very weak and almost undetectable (Figure 7C). The expression of *SsPAL1* was also induced by 4 °C low temperature, with the highest expression level after 4 h of treatment, and then declined to the initial level after 12 h treatment. When prolonging the treatment time to 24 h, its expression was decreased only 0.3-fold of the untreated control (Figure 7D). Along with the extension of treatment time, *SsPAL1* transcription levels were reduced by dark, but enhanced by light (Figure 7E,F). For wounding treatment, the expression level of *SsPAL1* was increased to the highest level at 6 h after treatment and then downregulated (Figure 7G). Under UV-B treatment, *SsPAL1* transcripts reached the highest level at 0.5 h and then gradually declined, until almost being undetectable at 8 h (Figure 7H).

These data confirmed that *SsPAL1* showed a rapid response in early stages of various stress conditions, which was consistent with the *cis*-acting elements in the 5′-flanking region of *SsPAL1* and coincided with previous reports on *S. miltiorrhiza* [15], *G. biloba* [29], *J. curcas* [32], *P. crispum* [36] and tobacco [37]. For example, in *S. miltiorrhiza*, the expression level of *SmPAL1* is strongly induced by light and wounding and a quick response to dehydration. Low temperature was another induction factor for enhancing the expression of PAL genes in *S. miltiorrhiza*, *G. biloba*, *J. curcas* and *A. thaliana*. These similar phenomena were also observed in some other species. On the other hand, the expression level of induction is different. For example, wounding treatment only moderately enhanced the expression of PAL gene in tobacco [37]. ABA treatment decreased significantly the expression of *AtPAL1* [15]. However, the expression of *SsPAL1* or *SmPAL1* was increased markedly under treatment with ABA. Taken together, the differences in expression level of induction depend on the stress and species of plant.

### 2.7. Expression of SsPAL1 in E. coli

**Figure 8:**
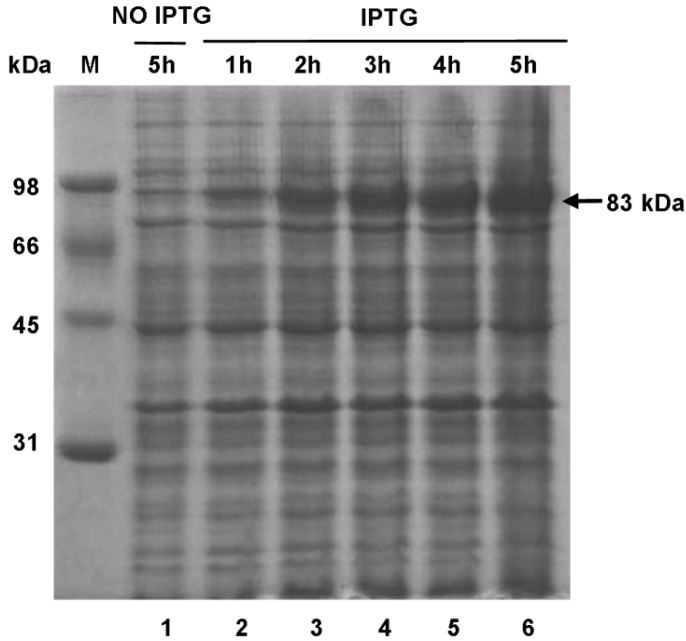
Expression of recombinant *SsPAL1* protein in *E. coli* strain BL21. SDS-PAGE patterns of total crude protein under non-induced and induced conditions with a time course, visualized by Coomassie Brilliant Blue R250 staining. Lane 1: *E. coli* strain BL21 harboring the expression plasmid pET30a-*SsPAL1* without induction by IPTG for 5 h; Lanes 2–6: *E. coli* strain BL21 harboring the expression plasmid pET30a-*SsPAL1* induced by IPTG for 1, 2, 3, 4 and 5 h, respectively. M, protein molecular mass marker; the arrow indicates the recombinant *SsPAL1* protein (including 6 KDa for tags).

**Figure 9:**
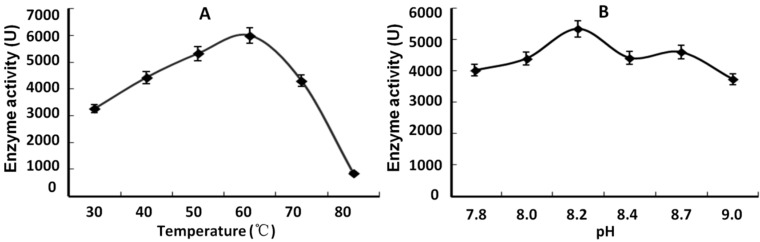
Characterization of recombinant *SsPAL1* expressed in *E. coli* BL21 (DE3). (**A**) Optimal temperature analysis of recombinant *SsPAL1*; (**B**) optimal pH analysis of recombinant *SsPAL1*. Data represent the mean values of three replicates (±SD).

The results of multiple sequence alignment and tertiary model analysis revealed that *SsPAL1* showed high sequence identity and structural similarity to other functional plant PAL proteins. To determine the functional activities of *SsPAL1*, the ORF was amplified and cloned into pET30 (+) using a previously described method [38], then expressed in the *E. coli* BL21 (DE3) strain, under the optimal incubation conditions of 28 °C, 5 h and 1 mM IPTG. SDS-PAGE analysis of total crude protein revealed that the recombinant *SsPAL1* was found mainly in the cytosol, and the molecular weight (including 6 KDa for tags) was about 83 kDa (Figure 8). Assessment of the enzyme activity of the crude protein extract showed that the recombinant *SsPAL1* had high catalytic activity to convert l-phenylalanine to trans-cinnamic acid. The optimal temperature and pH for expression of *SsPAL1* recombinant protein were 60 °C and 8.2, respectively (Figure 9A,B). The optimal temperature of the recombinant *SsPAL1* activity is the same as for *SmPAL1* and *JcPAL1* (60 °C) [15,32], close to PcPAL (58 °C) and *ZmPAL* (55–60 °C) [36,39], but higher than *AtPALs* (31–48 °C) [13] and *BoPALs* (50 °C) [17]. The optimal pH of the recombinant protein was 8.2, within the ranges known for plant PALs [13].

### 2.8. Correlation Analysis between PAL Activity and Anthocyanin Accumulation in Coleus

**Figure 10:**
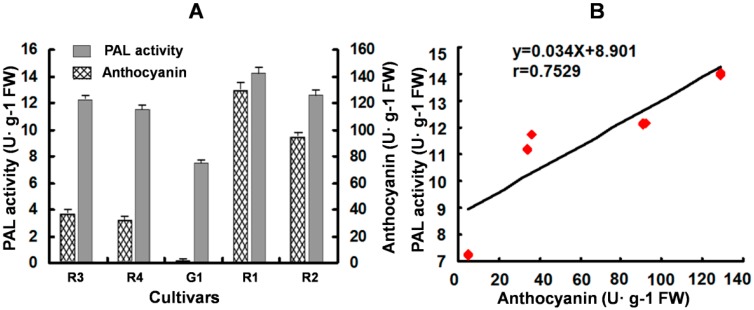
Analysis of *PAL* activity and anthocyanins concentration (**A**) and their correlation analysis (**B**) in the leaves of five *Coleus* cultivars. Each datum represents the average of three experiments (±SD) in (A). R1, R2, R3, R4 and G1 are different cultivars explained in Section 3.1.

As the first key enzyme in the phenylpropanoid pathway, the PAL activity could influence the products of different downstream branches. For identifying the effect on anthocyanin content, the relationship between the PAL activity and anthocyanins content in leaves of five *Coleus* cultivars were investigated. The highest level of PAL activity and anthocyanin accumulation was detected in the R1 cultivar with a dark red leaf color, and the lowest was found in G1 with a green leaf color. Despite there being wide variation in the level of PAL activity and anthocyanin content in different cultivars, their trend was observed to be similar (Figure 10A): the cultivar with a high level of PAL activity has high anthocyanin content and *vice versa*. However, further statistical analysis showed that there was no significant correlation between them (*r* = 0.7529, *p* > 0.1). This result suggested that PAL was not the rate-limiting enzyme for anthocyanin biosynthesis in *Coleus*. The influence of PAL activity on anthocyanins content is indirect in *Coleus*.

Similar relationships between PAL activity and anthocyanins accumulation have been reported in many plants, such as grapes [40], strawberries [41], apples [42,43] and litchi [44]. It is believed that PAL activity is more closely related to flavonoid biosynthesis than anthocyanins. In the absence of precursors for anthocyanin synthesis, changes in anthocyanin accumulation were mainly determined by the changes in PAL activity [26,43]. Plant PAL genes and many key genes of anthocyanin biosynthesis are co-expressed and induced by light stimulus [45]. In *Coleus* leaves, *SsPAL1* expression is enhanced by light (in this study), and the anthocyanin accumulation is also upregulated by light treatment [46]. The high-PAL enzyme activity leads to the production of a large number of precursors as substrates for anthocyanins biosynthesis. PAL activity and anthocyanin accumulation show a similar variation trend in different *Coleus* cultivars.

## 3. Experimental Section

### 3.1. Plant Material and Treatments

Five *Coleus* (*S. scutellarioides* (L.) Codd) cultivars (Figure 1), “Limelight” (named G1 (G, green)), “Red Trailing Queen” (named R1), “Copper Glow” (named R2), “Heart” (named R3) and “Rainbow mixed” (named R4), were grown in a greenhouse under natural light conditions at 25 °C.

R1 (dark red with chartreuse-green edges in leaf), R2 (light red leaf), R3 (yellow with dark red heart-shaped pattern in leaf), R4 (green with pink red heart-shaped pattern in leaf) and G1 (green with lemon yellow in leaf) were used to study gene expression in different organs. The one-month-old seedlings of R1 were treated with 100 μM ABA (abscisic acid), 500 μM SA (salicylic acid), dehydration, 4 °C cold, dark, light, wounding and UV-B, according to previous studies [16,29]. The details are as follows. For ABA and SA treatments, the roots of seedlings were soaked in a solution of certain concentration of hormones. Dehydration was performed on dry filter paper. Cold treatment was carried out in a 4 °C chamber. For light treatment, seedlings were grown in the chamber under strong light at 25 °C. The dark treatment was placing in a dark closed chamber at 25 °C. Wounding treatment was performed by cutting leaves of the one month-old seedlings. For UV-B treatment, seedlings were exposed under 1500 μJ/m^2^ UV-B irradiation in a dark closed chamber. All samples were collected after different treatments at different time points (shown in Figure 7) and then were immediately frozen in liquid nitrogen and stored at −80 °C until RNA isolation.

### 3.2. Cloning of the Full-Length SsPAL1

Genomic DNA was extracted from leaves of R1 using the modified CTAB method [47]. RNA was extracted from different organs of R1 and G1 and all samples of different treatments, by using TRIzol reagent (Invitrogen, Carlsbad, CA, USA), according to the protocol of the manufacturer, followed by incubation with RNase-free DNase I (Takara, Dalian, China), respectively. The quality and concentration of DNA and RNA were determined with electrophoresis and spectrophotometer.

Degenerate primers, FdPAL and RdPAL, were designed according to the conserved regions of plants *PAL* genes and used to amplify the cDNA fragment of *SsPAL* by DOP-PCR. Based on the conserved sequence of *PAL* genes, the 5′ and 3′ ends of *SsPAL1* were amplified by the RACE method, according to GeneRacer Kit’s protocol (Invitrogen, USA). In 5′ RACE, kit primers 5′P and 5′NP were paired with antisense primers PAL1-5′P and PAL1-5′NP for primary and nested amplifications, respectively. Sense primers PAL1-3′P and PAL1-3′NP were paired with kit primers 3′P and 3′NP for primary and nested amplifications of 3′ ends. The full-length cDNA and DNA was then amplified with two specific primers. All amplified products were purified, subcloned and sequenced. Primers used in this gene cloning are listed in Table S1.

The TD-PCR (touchdown) program was used for all PCR: 94 °C for 4 min, 20 cycles of amplification (94 °C for 30 s, 65 °C to 55 °C by −0.5 °C every cycle for 30 s, 72 °C for 2 min), 30 cycles of amplification (94 °C for 30 s, 55 °C for 30 s, 72 °C for 2 min), then 72 °C extension for 5 min.

### 3.3. Isolation of the 5′-Flanking Region of SsPAL1

To isolate the 5′-flanking region of *SsPAL1*, genome walking was performed by using the hiTAIL-PCR (high-efficiency thermal asymmetric interlaced PCR) [48]. Three gene-specific primers (GSP 1, GSP2 and GSP3; Table S1) were designed based on the 5′-end coding sequence of *SsPAL1* and paired with antisense primers of hiTAIL-PCR for three rounds of amplification. The amplification condition and the arbitrarily degenerative primers of hiTAIL-PCR were according to the previous report [48]. The target bands were subcloned and used for DNA sequencing.

### 3.4. Bioinformatics Analysis

The nucleotide and deduced protein sequence of *SsPAL1* and multiple alignments were analyzed by Vector NTI Suite 10.0 software (Invitrogen, Carlsbad, CA, USA). The phylogenetic tree of plant PALs was constructed by using MEGA 6.0 program. BLAST analysis was performed on the NCBI server [49]. Structural analysis of deduced protein was carried out on the website of Expasy Molecular Biology Server [50]. Homology-based structural modeling was performed by Swiss-Model [25] and shown with PyMol software. Prediction of the transcriptional start site and the putative *cis*-acting elements were performed using online server PLACE [51] and the Plant-CARE database [52].

### 3.5. Expression Analysis of SsPAL1 by Real-Time Quantitative RT-PCR

Real-time quantitative RT-PCRs (qRT-PCR) were performed to detect the expression of *SsPAL1* in different organs of five *Coleus* cultivars and treated leaves of R1. Total RNA was used as a template to generate first-strand total cDNA using the MMV kit (Promega, Madison, WI, USA). For qRT-PCR analyses, the transcript levels of the *SsPAL* gene in *Coleus* different tissues were measured. The *Coleus* actin gene, *SsACT* (GenBank Accession No. DQ423374), was used as an internal reference. One microliter of cDNA from each sample was used in a 20-μL PCR reaction volume, and the PCR conditions were: 2 min pre-denaturation at 94 °C, 1 cycle; 20 s denaturing at 94 °C, 20 s annealing at 60 °C, 25 s elongation at 72 °C, 35 cycles. The products of qRT-PCR were analyzed by 2% agarose gel electrophoresis and showed bands with predicted sizes. All qRT-PCR assays were performed using 2× SYBR Green Master Mix reagent (TaKaRa, Dalian, China), on a Bio-Rad IQ5 real-time PCR detection system. The results were calculated as differences in cycle threshold (Ct) between *SsPAL* and *SsACT* gene (2^−∆Ct^). All transcripts expressed in *Coleus* leaves treated by different conditions were analyzed using the comparative Ct method and quantified relative to the control (0 h, untreated leaves) (2^−∆∆Ct^). All qRT-PCR reactions were performed in triplicate and repeated twice. Statistical analysis was performed by SPSS software, and Tukey’s test was used to conduct pairwise comparison between means at a level of *p* < 0.05. The primer sequences for qRT-PCR are listed in Table S1.

### 3.6. Expression of SsPAL1 in Escherichia coli BL21 (DE3)

The coding sequence of *SsPAL1* was amplified by primers Fpal1 and Rpal1 (containing 5′-overlapping homologous region) (Table S1) and inserted into the lined pET30a (+) expression vector (Novagen, Madison, WI, USA) digested by *EcoR* I and *Hind* III using the plasmid isothermal assembly method [38]. Positive recombinant plasmid, pET30a-*SsPAL1*, was confirmed by sequencing and then transformed into the *E. coli* BL21 (DE3) strain for protein expression.

The transformants were inoculated at 37 °C in LB medium until OD_600_ reaching about 0.6. Then, a final concentration of 1 mM IPTG was added into the cultures to induce the protein expression at 28 °C. The cultivation was continued for 1, 2, 3, 4 and 5 h. The total protein, on 10% SDS-PAGE followed by staining with Coomassie Brilliant Blue R250, was used to assess the expression level of the induced protein.

### 3.7. Enzyme Activity Assay for SsPAL1

Cells harboring the *SsPAL1* protein were harvested by centrifugation at 8000 rpm for 15 min after 5 h of IPTG treatment at 28 °C and lysed by sonication in PBS buffer (50 mM NaH_2_PO_4_, pH 7.4). The lysate was centrifuged at 12,000 rpm for 15 min at 4 °C, and the resulting supernatant was collected for enzyme activity assay using a previously described method with minor modifications [53].

The reaction mixture was pre-incubated for 5 min prior to assay initiation by the addition of l-phenylalanine, while controls without the substrate. After 20 min, the reaction was terminated by incubating on ice. The *SsPAL1* activity was determined by measuring the absorbance of the formation of cinnamic acid at 290 nm in a reaction solution using a spectrophotometer. For the determination of optimum temperature, the reaction mixtures (containing 50 µL *SsPAL1* crude extract, 2 mL 0.01 M borate buffer and 1 mL 0.02 M l-phenylalanine, pH 8.2) were incubated at varying temperatures (30, 40, 50, 60, 70 and 80 °C). To determine the optimum pH, 0.01 M borate buffers were adopted with various pH (7.8, 8.0, 8.2, 8.4, 8.7 and 9.0).

### 3.8. PAL Activity and Anthocyanin Concentration in Coleus Cultivars

Leaves of five *Coleus* cultivars were used to analyze the potential relationship between anthocyanins and PAL activity. Anthocyanin concentration was assessed according to a previous report [54] with a little modification. In brief, anthocyanins were extracted from leaves with HCl/methanol (1:99, *v*/*v*) at 4 °C for 24 h in darkness, followed by centrifugation at 12,000 rpm for 20 min. For the supernatant, the absorbance of OD530 was measured as the anthocyanin content. One unit of anthocyanin equals 0.1 absorbance unit in 1 mL of extraction solution. Absorbance values were normalized to the fresh weight of the leaf samples.

Crude protein extract of leaves was used to assay the total PAL activity adopting the reported method [44]. Briefly, 0.5 g of leaves were homogenized in extraction buffer (0.05 M borate buffer, 0.05 M ascorbate and 0.018 M PVP, pH 7.0) on ice at 4 °C and then centrifuged at 12,000 rpm for 20 min. The supernatant of the crude extract of leaves was used to determine the enzyme activity of PAL according to Lister [26]. One unit of enzyme activity was defined as an increase in absorbance of 0.1 per hour per mL of the enzyme solution. PAL activity was expressed as enzyme units per gram of fresh weight (U·g^−1^ FW).

Statistical analysis using the SigmaPlot software was performed to determine the correlation between PAL activity and anthocyanin concentration in the leaves of five *Coleus* cultivars.

## 4. Conclusions

In this study, the first *Coleus PAL* gene, *SsPAL1*, was successfully isolated and characterized. The *SsPAL1* gene consisted of one intron and two exons. In its 5′-flanking region, there were many *cis*-acting elements involved in light and stress responsiveness, which was in accordance to the transcription patterns of *SsPAL1.* The expression levels of *SsPAL1* were enhanced by light and different abiotic stresses. Multiple sequence alignment and tertiary structure model analysis displayed that the deduced *SsPAL1* has highly-conserved active site, a high sequence identity and structural similarities with other functional plant PAL proteins. Further, the recombinant *SsPAL1* protein in *E. coli* strain BL21 exhibited high PAL activity with optimum temperature at 60 °C and optimum pH at 8.2, respectively. Furthermore, the total PAL activity was not significantly correlated with anthocyanin accumulation, suggesting that PAL was not the rate-limiting enzyme for anthocyanin biosynthesis in *Coleus*. Taken together, the isolated *SsPAL1* is a functional gene with typical molecular characteristics of plant PAL enzymes and might play an important role in phenylpropanoid biosynthesis in *Coleus*.

## Supplementary Materials

Supplementary materials can be accessed at: http://www.mdpi.com/1420-3049/20/09/16833/s1.
