# Supplementary material for: Isolation and Functional Characterization of a Phenylalanine Ammonia-Lyase Gene (SsPAL1) from Coleus (Solenostemon scutellarioides (L.) Codd)
Source: Molecules. 2015 Sep 16;20(9):16833–51. doi: 10.3390/molecules200916833 (PMC6332037; doi:10.3390/molecules200916833)
Supplement: Supplementary file 1 [file molecules-20-16833-s001.pdf]

Supplementary Materials

Table S1. List of PAL genes used in the phylogenetic tree analysis.

**Species**	**PAL Sequence Names**
**Bryophyta**	
Physcomitrella patens	Pp3c26_2420V1.1, Pp3c13_9000V1.1, Pp3c22_17540V1.1, Pp3c14_11870V1.1
**Gymnosperm**	
Pinus taeda	Pteda1143311, Pteda17307, Pteda28316, Pteda34319, Pteda9006
**Monocotyledons**	
Brachypodium distachyon	Bradi3g47110.1, Bradi3g49260.1, Bradi3g49270.1, Bradi3g49280.1, Bradi3g49250.2, Bradi5g15830.1, Bradi3g48840.1,Bradi3g47120.1
Oryza sativa	LOC_Os04g43760.1, LOC_Os02g41630.2, LOC_Os05g35290.1 LOC_Os12g33610.1, LOC_Os02g41650.3, LOC_Os02g41680.1 LOC_Os02g41670.1, LOC_Os11g48110.1
Sorghum bicolor	Sobic.001G160500.1, Sobic.004G220500.1, Sobic.004G220600.1, Sobic.004G220400.1, Sobic.004G220300.1, Sobic.004G220700.1, Sobic.006G148900.1
**Dicotyledons**	
Arabidopsis thaliana	AT2G37040.1(AtPAL1), AT3G53260.1(AtPAL2), AT3G10340.1(AtPAL4), AT5G04230.2(AtPAL3)
Cucumis sativus	Cucsa.137590.1, Cucsa.155520.1, Cucsa.385970.1, Cucsa.124500.1, Cucsa.124490.1 ,Cucsa.124510.1, Cucsa.124460.1, Cucsa.124450.1, Cucsa.124470.1
Glycine max	Glyma.03G181600.1, Glyma.19G182300.1, Glyma.10G058200.1, Glyma.10G209800.1, Glyma.02G309300.1, Glyma.20G180800.1,
Populus trichocarpa	Potri.010G224200.1, Potri.010G224100.1, Potri.006G126800.1, Potri.008G038200.1
**Lamiales**	
Solenostemon scutellarioides	*SsPAL1* (JQ975419)
Sesamum indicum	*SiPAL* (XP_011077338.1)
Salvia miltiorrhiza	*SmPAL* (AGW27206.1)
Scutellaria viscidula	*SvPAL* (ACR56688)
Pogostemon cablin	*PcPAL* (AJO53274)

Note: The PAL genes identified from Phytozome 10.2V database (http://phytozome.jgi.doe.gov/pz/portal.html) and NCBI.

Table S2. List of primers used in this study.

Primer Name	Sequence	Note
FdPAL	5′-GCHTCBGGTGATYTRGTY-3′	DOP-PCR for conserved fragment of PAL
RdPAL	5′-ACATCTTGGTTRTGYTGCTC-3′	fragment of PAL
PAL1-5′P	5′-CGGCGAAGATTGCTGAAATCACAACGG-3′	For 5′-RACE
PAL1-5′NP	5′-CTTGAAAGCCTCCTCGGCGTTGAGCTC-3′	
PAL1-3′P	5′-GCTCAATTCTCCGAACTCGTCAACGAC-3′	For 3′-RACE
PAL1-3′NP	5′-CCCTCCAACCTCTCCGGTGGGAGGAAC-3′	
Fpal1	5′-<u>ATGGCTGATATCGGATCCGAATTC</u>ATGGCGG CAGCGACGGAAAACG-3′	For amplifying full-length ORF of cDNA and DNA
Rpal1	5′-<u>CTCGAGTGCGGCCGCAAGCTT</u>GACATATTGG AAGAGGTTCACC-3′	For *E. coli* expression vector construction
GSP1	5′-TTCCGTCGCTGCCGCCATGATC-3′	For hiTAIL-PCR
GSP2	5′-GCCATGATCGGATTTTAAACCGGTGGAG-3′	
GSP3	5′-GGATTTTAAACCGGTGGAGATGATGGCAAG-3′	
SsPAL1-Fq	5′-GCCAGCAGTGATTGGGTTAT-3′	For qPCR expression analysis
SsPAL1-Rq	5′-CAAGTTTCCGTCCCTTTTCC-3′	
SsACT-Fq	5′-GGCTTACACCATCACCAGAGT-3′	For qPCR expression analysis as a inter control
SsACT-Rq	5′-CCAAGGCGAACAGAGAGAAA-3′	

Note: Underlined bases correspond to overlapping sequences with multiple cloning sites of pET30a (+).

Table S3. Function of the *cis*-elements in 5′- flanking region of *SsPAL1*.

***Cis*-Element**	**Function**
TATA-box	Core promoter element around −30 of transcription start
CAAT-box	CAAT-box common cis-acting element in promoter and enhancer regions
L-Box	One of three putative cis-acting elements of phenylalanine ammonia-lyase genes
P-Box	One of three putative cis-acting elements of phenylalanine ammonia-lyase genes
MYBP	MYB binding site involved in light responsiveness and related to P- box
MYB core	MYB binding site involved in regulation of flavonoid biosynthesis
MBS	MYB binding site involved in drought-inducibility
G-Box	*Cis*-acting regulatory element involved in light responsiveness
ABRE	*Cis*-acting element involved in the abscisic acid responsiveness
CAT-box	*Cis*-acting regulatory element related to meristem expression
CTAG-motif	Critical de-terminant for repressor binding
GAG-motif	Part of a light responsive element
GARE-motif	Gibberellin-responsive element
GC-motif	Enhancer-like element involved in anoxic specific inducibility
GCN4_motif	*Cis*-regulatory element involved in endosperm expression
Skn-1_motif	*Cis*-acting regulatory element required for endosperm expression
rbcS-CMA7	Part of a light responsive element
TGACG-motif	*Cis*-acting regulatory element involved in the MeJA-responsiveness
W1-box	WRKY binding site involved in defense, wounding and pathogen responsiveness

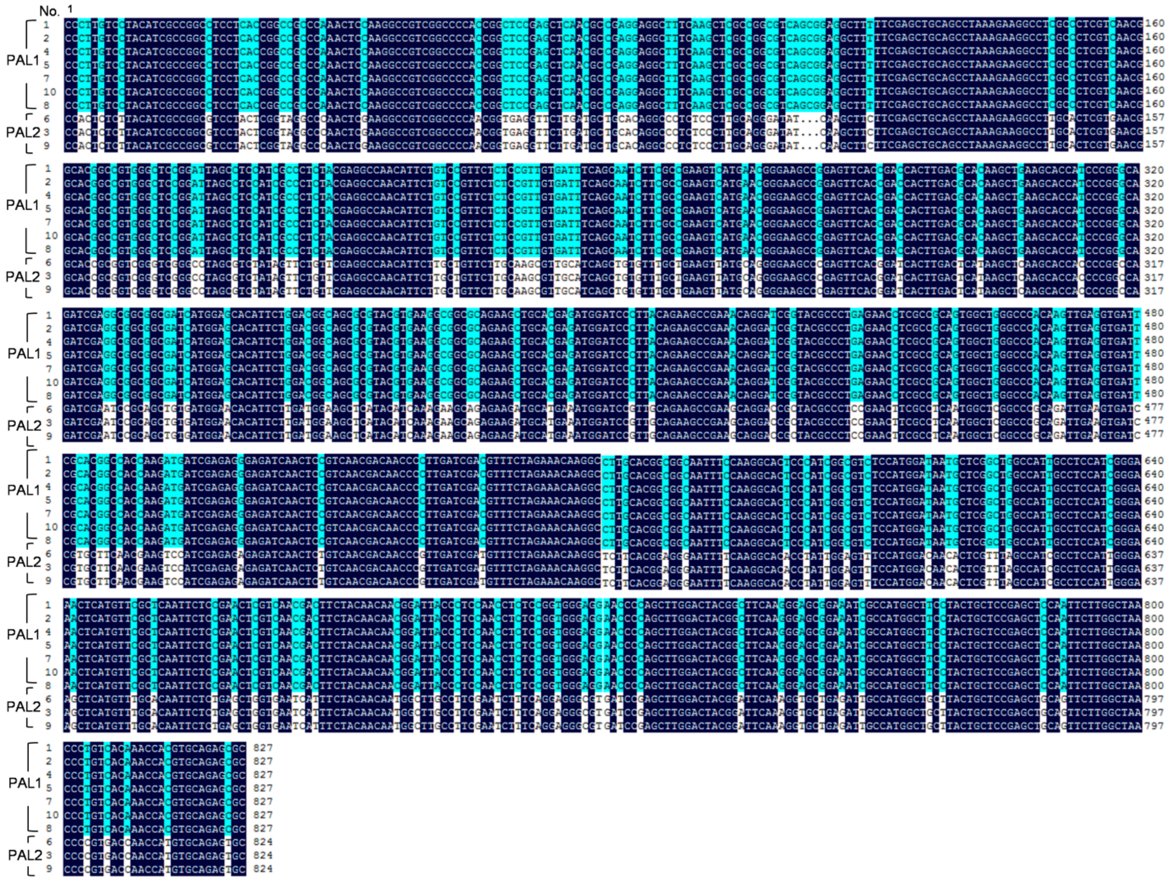

Figure S1. Alignment of conserved coleus PAL gene fragments from 10 clones.

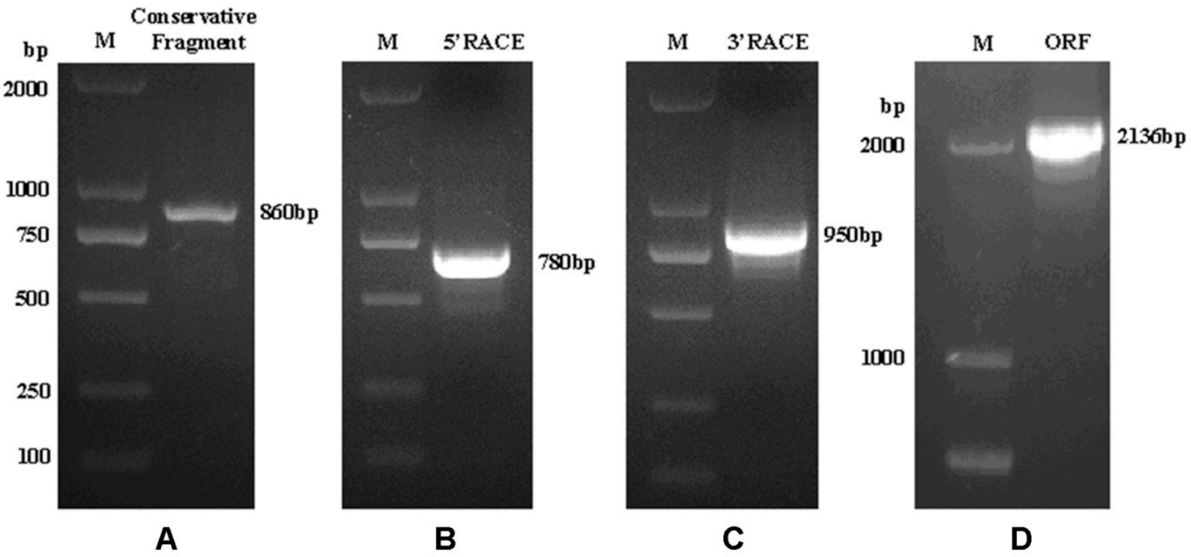

Figure S2. Molecular cloning of *SsPAL1*. (**A**) Comservative fragment amplification of *SsPAL1* gene by DOP-PCR; (**B**) 5′ RACE; (**C**) 3′ RACE; (**D**) ORF full-length amplification.

```
   1 ATGGCGGCAGCGACGGAAAACGGTCACCAATCCAACGGGTTCTGCGTGAAGCAGAACGACCCACTGAATTGGGCAGCGGCGGCGGAGGCGCTGCAG
      M  A  A  A  T  E  N  G  H  Q  S  N  G  F  C  V  K  Q  N  D  P  L  N  W  A  A  A  A  E  A  L  Q   32
  97 GGGAGCCATCTCGATGAGGTGAAGAAGATGGTGGAGGAGTTCAGGAAGCCGGCTGTGAAACTCGGCGGAGAGACTCTGACTATTGCCCAGGTGGCG
      G  S  H  L  D  E  V  K  K  M  V  E  E  F  R  K  P  A  V  K  L  G  G  E  T  L  T  I  A  Q  V  A   64
 193 GCGATCGCGGCCAGGGACAATGCGGTAGCGGTTGAGCTGTCTGAGGCGGCGAGGGCCGGCGTGAAAGCCAGCAGTGATTGGGTTATGGAAAGCATG
      A  I  A  A  R  D  N  A  V  A  V  E  L  S  E  A  A  R  A  G  V  K  A  S  S  D  W  V  M  E  S  M   96
 289 AACAAAGGAACCGACAGCTACGGCGTCACCACCGGCTTCGGTGCCACGTCTCACCGGAGAACTAAGCAGGGCGGCGCTCTGCAGACGGAGCTGATC
      N  K  G  T  D  S  Y  G  V  T  T  G  F  G  A  T  S  H  R  R  T  K  Q  G  G  A  L  Q  T  E  L  I  128
 385 AGGTAATTAATTTATTGCAAAAAATCATTATTTTTCCATAATCGGAAACTAATCCTAAATCTCAGTAATTATTAAATTGACAATAATAATAATAGG
      R -----------------------------------------intron------------------------------------------ 129
 481 ATCCACGTGGTAAGAGGTAGAAAATATTTGAATATTTATGAAATAAAATAGATATATTTATTGGAATATATGATGATGGATTGGATCTGAAACAGA
     ----------------------------------------------------------------------------------------------
 577 TTTCTAAACGCCGGAATATTCGGAAAAGGGACGGAAACTTGCCACACCCTACCGCACTCGGCGACGAGGGCAGCAATGCTTGTTAGAATCAACACT
      F  L  N  A  G  I  F  G  K  G  T  E  T  C  H  T  L  P  H  S  A  T  R  A  A  M  L  V  R  I  N  T  161
 673 CTTCTTCAGGGATATTCCGGCATCAGATTCGAAATCTTGGAAGCCATTACCAAATTCCTCAACCACAACATCACTCCCTGCCTCCCCCTCCGCGGC
      L  L  Q  G  Y  S  G  I  R  F  E  I  L  E  A  I  T  K  F  L  N  H  N  I  T  P  C  L  P  L  R [G] 193
 769 ACCATCACCGCCTCCGGTGATCTCGTTCCCTTGTCCTACATCGCCGGCCTCCTCACCGGCCGCCCAAACTCCAAGGCCGTCGGCCCCACCGGCTCC
    [ T  I  T  A  S  G  D  L  V  P  L  S  Y  I  A  G] L  L  T  G  R  P  N  S  K  A  V  G  P  T  G  S  225
 865 GAGCTCAACGCCGAGGAGGCTTTCAAGCTCGCCGGCGTCAGCGGAGGCTTTTTCGAGCTGCAGCCTAAAGAAGGCCTCGCCCTCGTCAACGGCACG
      E  L  N  A  E  E  A  F  K  L  A  G  V  S  G  G  F  F  E  L  Q  P  K  E  G  L  A  L  V  N  G  T  257
 961 GCCGTGGGCTCCGGATTAGCCTCCATCGCCCTCTACGAGGCCAACATTCTGTCCGTTCTCTCCGTTGTGATTTCAGCAATCTTCGCCGAAGTCATG
      A  V  G  S  G  L  A  S  I  A  L  Y  E  A  N  I  L  S  V  L  S  V  V  I  S  A  I  F  A  E  V  M  289
1057 AACGGGAAGCCGGAGTTCACCGACCACTTGACGCACAAGCTGAAGCACCATCCCGGGCAGATCGAGGCGGCGGCGATCATGGAGCACATTCTGGAC
      N  G  K  P  E  F  T  D  H  L  T  H  K  L  K  H  H  P  G  Q  I  E  A  A  A  I  M  E  H  I  L  D  321
1153 GGCAGCGCGTACGTGAAGGCGGCGCAGAAGCTGCACGAGATGGATCCCTTACAGAAGCCGAAACAGGATCGGTACGCCCTGAGAACCTCGCCGCAG
      G  S  A  Y  V  K  A  A  Q  K  L  H  E  M  D  P  L  Q  K  P  K  Q  D  R  Y  A  L  R  T  S  P  Q  353
1249 TGGCTGGGCCCACAAGTTGAGGTGATTCGCACGGCCACCAAGATGATCGAGAGGGAGATCAACTCCGTCAACGACAACCCCTTGATCGACGTTTCT
      W  L  G  P  Q  V  E  V  I  R  T  A  T  K  M  I  E  R  E  I  N  S  V  N  D  N  P  L  I  D  V  S  385
1345 AGAAACAAGGCCTTGCACGGCGGCAATTTCCAAGGCACTCCCATCGGCGTCTCCATGGATAATGCTCGGCTGGCCATTGCCTCCATCGGGAAACTC
      R  N  K  A  L  H  G  G  N  F  Q  G  T  P  I  G  V  S  M  D  N  A  R  L  A  I  A  S  I  G  K  L  417
1441 ATGTTCGCTCAATTCTCCGAACTCGTCAACGACTTCTACAACAACGGATTACCCTCCAACCTCTCCGGTGGGAGGAACCCCAGCTTGGACTACGGC
      M  F  A  Q  F  S  E  L  V  N  D  F  Y  N  N  G  L  P  S  N  L  S  G  G  R  N  P  S  L  D  Y  G  449
1537 TTCAAGGGAGCGGAAATCGCCATGGCTTCCTACTGCTCCGAGCTCCAATTCTTGGCTAACCCTGTCACAAACCACGTGCAGAGCGCGGAGCAGCAC
      F  K  G  A  E  I  A  M  A  S  Y  C  S  E  L  Q  F  L  A  N  P  V  T  N  H  V  Q  S  A  E  Q  H  481
1633 AACCAAGATGTGAACTCCTTGGGGCTGATCTCGTCAAGGAAAACAGTCGAGGCATTGGATATCTTGAAGCTCATGTCTTCCACATTCCTCATCGCG
      N  Q  D  V  N  S  L  G  L  I  S  S  R  K  T  V  E  A  L  D  I  L  K  L  M  S  S  T  F  L  I  A  513
1729 CTGTGCCAAGCCATTGATTTGAGGCATTTGGAGGAGAATCTGAGACTGGCTGTGAAGAACACGGTGAGCCAAGTGGCGAAGAGAACTCTAACAATG
      L  C  Q  A  I  D  L  R  H  L  E  E  N  L  R  L  A  V  K  N  T  V  S  Q  V  A  K  R  T  L  T  M  545
1825 GGCATCAACGGCGAGCTCCATCCATCCAGATTCTGCGAGAAGGACCTCCTCCGCGTGGTGGACCGTGAGTACGTGTTCGCCTACATCGACGACCCC
      G  I  N  G  E  L  H  P  S  R  F  C  E  K  D  L  L  R  V  V  D  R  E  Y  V  F  A  Y  I  D  D  P  587
1921 TGCAGCGCCAACTACCCATTGATGCAGAAGCTGAGGCAGGTTTTGGTCGACCACGCCTTGAAAAATGGAGACAGTGAGAAGAATGTGGGCACTTCC
      C  S  A  N  Y  P  L  M  Q  K  L  R  Q  V  L  V  D  H  A  L  K  N  G  D  S  E  K  N  V  G  T  S  619
2017 ATCTTCCAGAAGATCCAGGTGTTCGAGGAGGAGCTTACGGCCGTCCTACCTAAGGAAGTGGAGGCCGCCAGAATCGCCTTGGAGGCCGGAAATCCG
      I  F  Q  K  I  Q  V  F  E  E  E  L  T  A  V  L  P  K  E  V  E  A  A  R  I  A  L  E  A  G  N  P  651
2113 GCGATTCCCAACAGGATCAAGGAATGCAGGTCGTATCCGTTGTACAGCTTCATCAGGGAGGAGTTGGGGACGGAGTTCTTGACGGGGGAGAAGACG
      A  I  P  N  R  I  K  E  C  R  S  Y  P  L  Y  S  F  I  R  E  E  L  G  T  E  F  L  T  G  E  K  T  683
2209 GTTTCGCCTGGAGAAGAGGGTGAGAAGGTGTTCACTGCTTTGAGCAATGGGTTGATTGTTGATCCGTTGTTGAAATGCCTTGAGGCTTGGAATGGT
      V  S  P  G  E  E  G  E  K  V  F  T  A  L  S  N  G  L  I  V  D  P  L  L  K  C  L  E  A  W  N  G  715
2305 GAACCTCTTCCAATATGTTAATTAGTTTTACTGGATTTTGTATGTGGTGATTGGTGATGGATGAATTGTATTGTAAAGAATTTCTTTATAATATGT
      E  P  L  P  I  C  *                                                                             721
2401 TCAAAATATAATTCTTAATCACAAAAAAAAAAAAAAAAAAAAAAAAAAAAA
```

Figure S3. The nucleotide sequence and the deduced amino acid sequence of *SsPAL1*. The start codon (ATG) and stop codon (TAA) are underlined. The putative polyadenylation signal (AATATAA) is italic, and active sites are in bold. The active site consensus sequence of phenylalanine and histidine ammonia-lyase is boxed. GT/AG splicing site is shaded. The MIO domain (N19-G256), Core domain (T257-H522, N645-C711), and Inserted shielding domain (L523-P644) are underlined, shaded and dot- underlined, respectively.
